# Retrospective analysis of 14 cases of disseminated *Penicillium marneffei* infection with osteolytic lesions

**DOI:** 10.1186/s12879-015-0782-6

**Published:** 2015-02-06

**Authors:** Ye Qiu, Jianquan Zhang, Guangnan Liu, Xiaoning Zhong, Jingmin Deng, Zhiyi He, Bai Jing

**Affiliations:** Department of Integrated Medicine, The Affiliated Tumor Hospital of Guangxi Medical University, Nanning, Guangxi China; Department of Respiratory Medicine, The First Affiliated Hospital of Guangxi Medical University, Nanning, Guangxi 530021 China

**Keywords:** HIV-negative, Penicilliosis marneffei, Ostealgia, Osteolytic lesion

## Abstract

**Background:**

*Penicillium marneffei* disseminates hematogenously and can infect most organs, though infection leading to osteolysis is extremely rare. We describe the clinical and laboratory features, management, and outcomes of patients with penicilliosis marneffei (PSM) with osteolytic lesions.

**Methods:**

This retrospective study was conducted between January 1, 2003 and May 1, 2014 at the First Affiliated Hospital of Guangxi Medical University. Patients who presented with culture and/or histopathologic proof of disseminated PSM within osteolytic lesions were included.

**Results:**

*P. marneffei* infection was diagnosed in 100 patients (65 HIV-infected and 35 HIV-negative). Fourteen patients, all HIV-negative, (14/35, 40%) had osteolytic lesions. The most common comorbidity was diabetes mellitus, though previous glucocorticoid therapy, β-thalassemia, breast cancer, and Langerhans cell histiocytosis also occurred. Five patients had no comorbidity. Fever, malaise, ostealgia, weight loss, and anemia were the most common symptoms, followed by cutaneous lesions, lymphadenopathy, hepatosplenomegaly, cough, sputum, and stethalgia. Ostealgia, joint pain, and joint disorders were also recorded. White blood cell and neutrophil counts were increased (mean 22.3 ± 7.4 × 10^9^ cells/L; mean 18.84 ± 4.5 × 10^9^ cells/L, respectively). The most common sites were the vertebrae, skull and femur, ribs and ilium, though the clavicle, scapula, humerus, and tibia were also involved. Radiography and computed tomography (CT) showed multiple radiolucencies with moth-eaten bone destruction, periosteal proliferation, bone fracture, and surrounding soft-tissue swelling. Emission CT showed significantly increased uptake in many skeletal regions. Positron emission tomography/CT showed generalized lymphadenopathy, bone metabolic activity, and bone destruction. The ^18^ F-FDG standard uptake value was increased in the entire skeleton (mean 6.16). Twelve patients received antifungal therapy, four of whom died during treatment, and eight recovered, though four of these eight relapsed within 3–24 months. Two patients discontinued treatment because of severe multiple organ failure and died.

**Conclusions:**

Osteolysis is often overlooked in HIV-negative individuals with disseminated *P. marneffei* infection. However, *P. marneffei* involving the bone and leading to osteolysis may indicate severe systemic disturbance, and is characterized by a poor prognosis, high recurrence rate, and the need for prolonged antifungal treatment.

## Background

Penicilliosis marneffei (PSM) is a deep fungal infection caused by *Penicillium marneffei*, and one of the most common opportunistic infections among patients with AIDS in Southeast Asia [1]. PSM is categorized as either localized or disseminated. Disseminated PSM usually affects the monocyte-macrophage system in multiple organs including the skin, lungs, and the reticuloendothelial system [2], while *P. marneffei* infection of the bone leading to osteolytic lesions is extremely rare [3]. In this study, we retrospectively analyzed the medical records and clinical characteristics of 14 patients diagnosed with disseminated PSM with osteolytic lesions between January 1, 2003 and May 1, 2014, at the First Affiliated Hospital of Guangxi Medical University. We describe the clinical and laboratory features, management, and outcomes of these patients to provide evidence for the correct diagnosis and early treatment of this condition.

## Methods

### Medical records

The medical records of 100 patients diagnosed with PSM between January 1, 2003 and May 1, 2014, at the First Affiliated Hospital of Guangxi Medical University were reviewed. Among these, 14 patients with osteolytic lesions were retrospectively evaluated. Patient information, including demographics (sex, age, and occupation), medical history (present history, comorbidities, and previous therapy), auxiliary examination results (hematological tests, serological tests, immune status, imaging examinations, and pathological and microbiological tests), and treatment, was collected from medical records and summarized for analysis.

This study was approved by the First Affiliated Hospital of Guangxi Medical University Signation Ethical Committee. All patients provided written informed consent.

### Diagnostic criteria for disseminated PSM

*P. marneffei* was isolated from clinical specimens including blood, bone marrow, bone, sputum, lung, or discharge from skin lesions and cultured on Sabouraud dextrose agar (SDA) at 25°C and 37°C. Positive *P. marneffei* cultures were characterized by dimorphic fungi that grew as a mold at 25°C and as a yeast at 37°C [4]. *P. marneffei* was identified based on cytology and histopathology from tissues and secretions stained with periodic acid-Schiff stain or Wright–Giemsa stain, by light microscopy. The yeast form of *P. marneffei* has a characteristic morphology including a transverse septum [5].

### Diagnostic criteria for PSM with osteolytic lesion

Patients were diagnosed according to the following criteria: (i) *P. marneffei* identified in bone and/or bone marrow biopsy samples using histopathology, cytologic smears, and fungal culture; (ii) disseminated PSM with osteolytic lesions diagnosed based on the presence of osteolytic lesions on imaging examination, clinical symptoms including ostealgia, improvement after receiving antifungal treatment alone, and exclusion of other diseases that cause osteolysis (tuberculosis, cancer, hematological diseases, and other fungal infections such as African histoplasmosis, blastomycosis, cryptococcosis, and coccidioidomycosis).

## Results

### Demographic data and clinical characteristics

During the 11-year study period, 100 patients were diagnosed with disseminated PSM according to the inclusion criteria, including 65 HIV-infected and 35 HIV-negative patients. Of these, only 14 patients, all HIV-negative, had osteolytic lesions and were enrolled. The incidence of *P. marneffei* with osteolytic lesions in HIV-negative patients was therefore 40% (14/35). The study population included nine men and five women with a median age of 42.17 years (range 22–67 years). Their occupations included farmer (n = 8), laborer (n = 3), cadre (n = 2), and student (n = 1). HIV-negativity was determined as negative for interferon-γ autoantibody using repeated enzyme-linked immunosorbent assay (ELISA) testing. Comorbidities included diabetes mellitus (n = 4), previous glucocorticoid therapy (n = 2), β-thalassemia (n = 1), breast cancer (n = 1), Langerhans cell histiocytosis (n = 1), and no comorbidities (n = 5). Fever, malaise, ostealgia, weight loss, and anemia were the most frequent symptoms, followed by cutaneous lesions, lymphadenopathy, hepatosplenomegaly, cough, sputum, and stethalgia. Ostealgia, joint pain, and joint disorders were also present (Table 1).

**Table 1:** **Clinical manifestations in patients infected with** ***Penicillium marneffei*** **with osteolysis (n = 14)**

**Symptom/sign**	**Patients**	
	**No.**	**%**
Fever	14	100
Malaise	14	100
Weight loss	13	92.85
Anaemia	13	92.85
Ostealgia	13	92.85
Cutaneous lesions	12	85.71
Lymphadenopathy	11	78.57
Hepatosplenomegaly	10	71.43
Cough and sputum production	10	71.43
Stethalgia	9	64.28
Dyspnea	5	35.71
Hemoptysis	2	14.28

### Laboratory examination

Complete blood count examinations revealed increased white blood cell levels in 12 patients (85.71%) (mean 22.3 ± 7.4 × 10^9^ cells/L, range 13.3–35 × 10^9^ cells/L). The hemoglobin concentration was decreased in 13 patients (92.85%) (mean 81.71 ± 3.2 g/L, range 65–116 g/L). Neutrophils were increased in 13 patients (92.85%) (mean 18.84 ± 4.5 × 10^9^ cells/L, range 8.8–28.2 × 10^9^ cells/L). The CD4 and CD8 lymphocyte counts were determined in 12 patients by flow cytometry and were decreased in nine patients (75%). Serum biochemical analysis revealed a mean serum albumin concentration of 20.1 ± 3.2 g/L below the normal range in 13 patients (92.85%, range 11.0–31.4 g/L). Six patients (42.85%) showed kidney dysfunction. C-reactive protein concentration and erythrocyte sedimentation rate were increased in all patients.

### Chest radiography and computed tomography

Chest computed tomography (CT) indicated pulmonary consolidation in three patients (21.43%), cavities in five (35.7%), disseminated inflammation in 12 (85.71%), and interstitial disease involving the pleura in two (14.28%). Ten (71.43%) patients had mediastinal and/or/ hilar lymphadenopathy, and nine (64.28%) had pleural inflammatory reaction and/or pleural effusion. Four (28.57%) patients had pericardial effusion, and three (21.43%) had seroperitoneum.

### Skeletal localization

In the 14 patients, *P. marneffei* infection involved multiple bony structures as follows: bones of the trunk (n = 11, 78.57%), limbs (n = 10, 71.43%), skull (n = 6, 42.85%), and cartilage (n = 1, 7.14%). The most common sites were the vertebrae (n = 10, 71.43%), skull and femur (n = 6, 42.85%), and ribs (n = 5, 35.71%). The ilium was affected in four (28.57%) patients, the clavicle, scapula, humerus, and tibia in three (21.42%), the radius, sternum, innominatum, fibula, and bones of the hand in two (14.28%), and the elbow joint, ischium, pubis acetabulum, bones of the foot, and the sacrum were implicated in one (7.14%) patient. Five (35.71%) patients showed joint involvement. Soft-tissue inflammation was identified surrounding the bone in seven (50%) patients (Table 2).

**Table 2:** **Skeletal localization in patients infected with** ***Penicillium marneffei*** **with osteolysis (n = 14)**

**Types of bones and site**		**Patients**	
		**No.**	**%**
Skull	6	42.85	
Bones of the trunk	Vertebrae	10	71.43
	Ribs	5	35.71
	Sternum	2	14.28
Bones of limbs	Femur	6	42.85
	Ilium	4	28.57
	Clavicle	3	21.42
	Scapula	3	21.42
	Humerus	3	21.42
	Shinbone	3	21.42
	Radial,	2	14.28
	Sternum,	2	14.28
	Innominatum,	2	14.28
	Fibula	2	14.28
	Bones of hand	2	14.28
	Elbow-bone	1	7.14
	Ischium	1	7.14
	Pubesacetabulum	1	7.14
	Bones of foot	1	7.14
	Sacrum	1	7.14
Cartilage	1	7.14	
Joint involvement	5	35.71	
Soft tissue swelling	7	50	

### Imaging examinations

**Figure 1:**
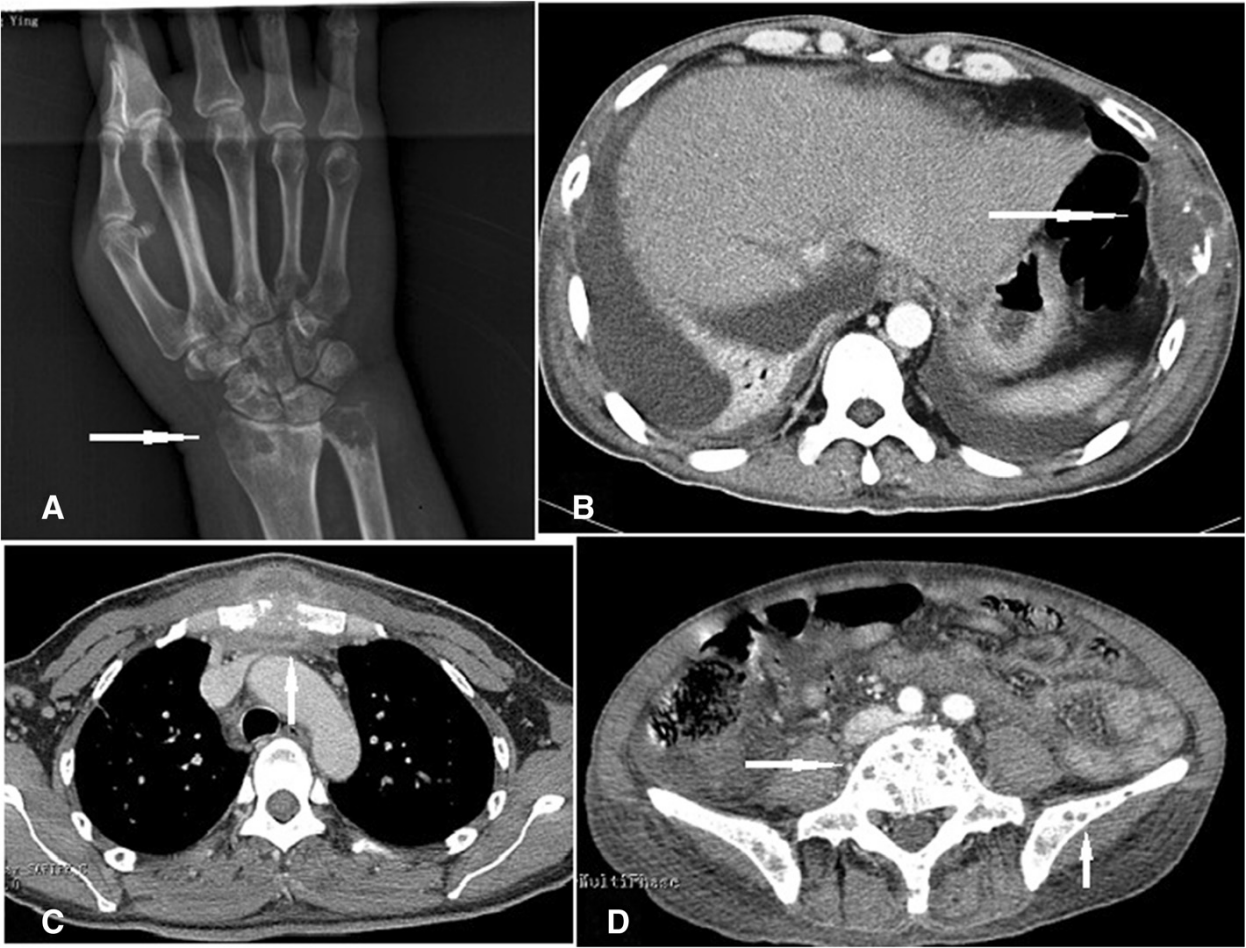
**Radiography and computed tomography showing osteolytic lesions characterized by multiple lucent defects with a moth-eaten destruction of bone in the head of the radius (A), ulna (A), rib (B), sternum (C), vertebrae (D), and ilium (D), and swelling in surrounding soft tissues (B, C).**

**Figure 2:**
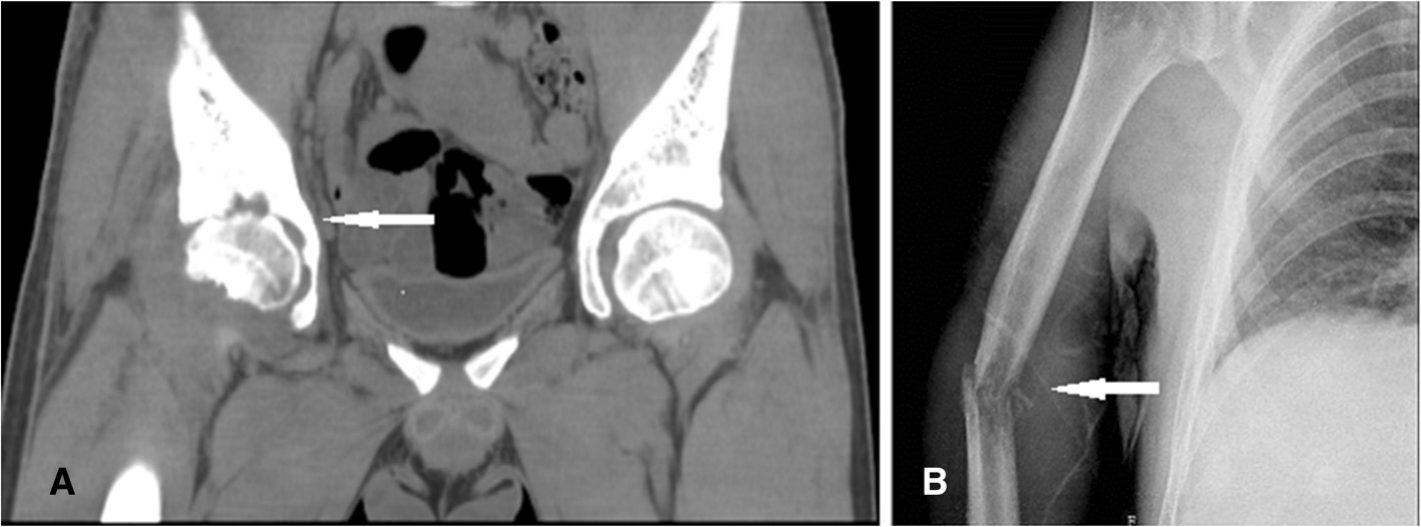
**Radiography and computed tomography showing periosteal proliferation in the innominatum and femur (A), and a pathologic fracture (B) in the humerus.**

**Figure 3:**
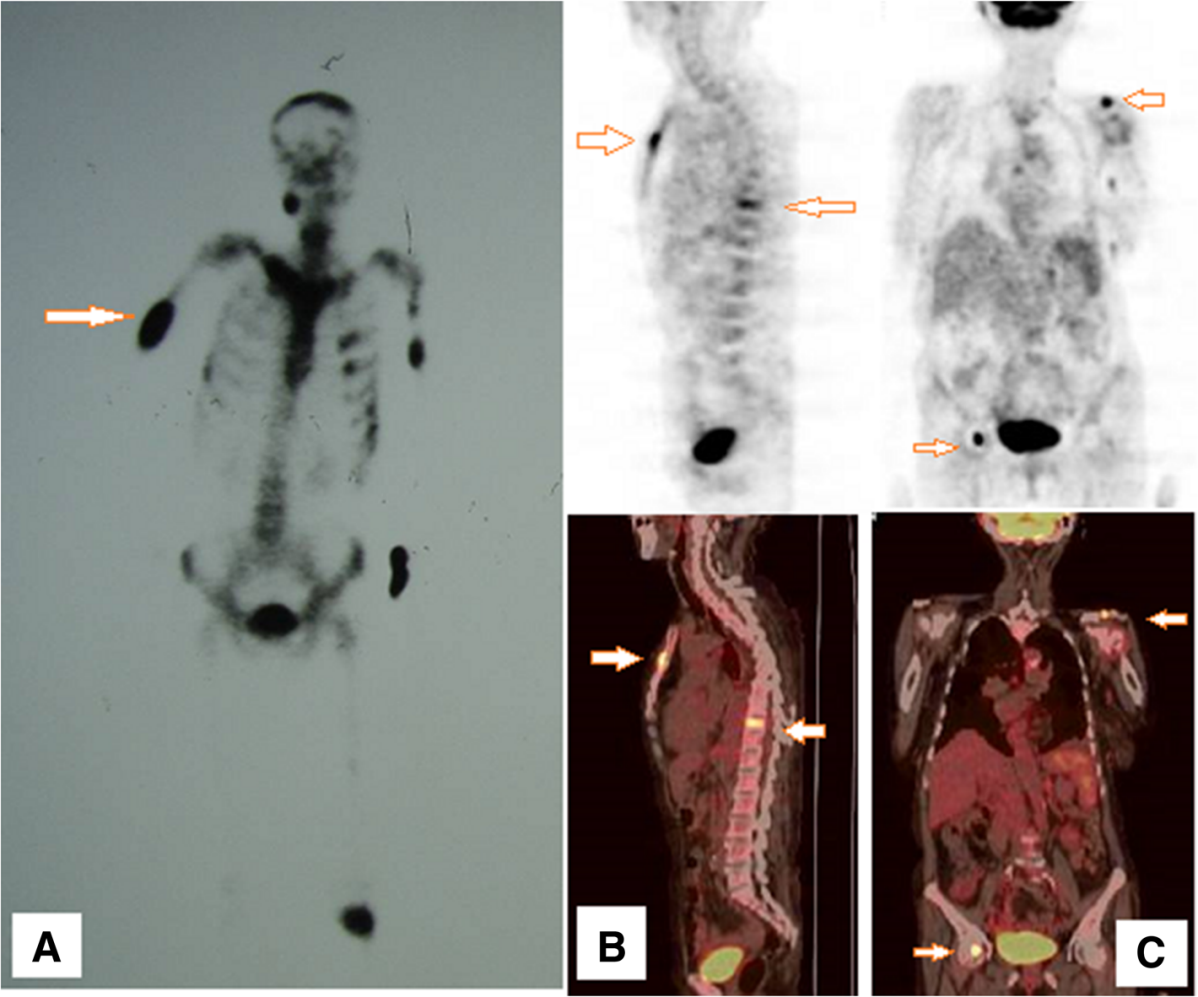
**Emission computed tomography (CT) showing increased radioactive concentration in the brustwirbels, sternum, ilium, and left clavicle (A).** Positron emission tomography/CT showed generalized lymphadenopathy, increased whole-body bone metabolic activity, and generalized skeletal destruction. The ^18^ F-FDG standard uptake value was increased in the whole skeleton and was 5.1 in the sternum **(B)**, and 6.1 in the femur and clavicle **(C)**.

Radiographic and CT examinations showed multiple radiolucencies, resembling a moth-eaten pattern of bone destruction (Figure 1) in eight cases (57.14%). Periosteal proliferation (Figure 2A) was identified in two cases (14.28%), fracture (Figure 2A and B) in six cases (42.85%), and surrounding soft-tissue swelling (Figure 1C) in five cases (35.71%). Emission CT (ECT) was performed in five cases and showed significantly increased uptake in multiple bones (Figure 3). Positron emission tomography/CT (PET/CT) was performed in two cases and showed diffuse lymphadenopathy, whole-body bone metabolic activity, and destruction in multiple bones. The ^18^ F-FDG standard uptake value (SUV) was increased in the entire skeleton (mean 6.16, range 2.5–9.6).

### Fungal culture and histopathology

**Figure 4:**
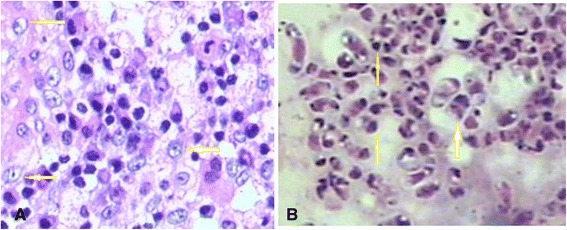
**Histopathology of cervical vertebral (periodic acid-Schiff stain, 400×) (A) and bone marrow samples.** Numerous intracellular yeast-like or sausage-like cells measuring 2–3 μm in diameter with a transverse septum were observed (arrows, periodic acid-Schiff staining, 1000×) **(B)**.

Fluid was aspirated from the bone marrow, blood, pleural effusion, seroperitoneum, subcutaneous abscess, skin ulcer, and dermal secretions, inoculated onto SDA, and incubated at 37°C and 25°C. Twelve cases were confirmed by positive *P. marneffei* culture. *P. marneffei* was isolated from sputum samples (1/14, 7.14%), venous blood (3/14, 21.42%), bone marrow (2/14, 14.28%), bronchoalveolar lavage fluid (1/14, 7.14%), pus from a subcutaneous abscess (3/14, 21.42%) and dermal lesion secretions (3/14, 21.42%). In addition, seven cases were diagnosed with *P. marneffei* infection by histopathology or cytology of specimens obtained from pulmonary (1/14, 7.14%), lymph node (2/14, 14.28%), bone marrow (2/14, 14.28%, Figure 4A), bone (1/14, 7.14%, Figure 4B), and skin lesions (2/14, 14.28%).

### Treatment and outcome

Twelve patients received intravenous amphotericin B deoxycholate (0.6–1.0 mg/kg/day) followed by oral itraconazole (400 mg/day). There was no consistent duration of antifungal therapy. Twelve patients received antifungal therapy, four of whom died during treatment. The other eight patients recovered, four of whom showed no recurrence, but the other four experienced a relapse 3–24 months later during follow-up. One of these patients received intravenous amphotericin B deoxycholate followed by itraconazole antifungal treatment for 18 months, but relapsed 6 months after discontinuing treatment. Antifungal treatment was discontinued in two patients because of severe multiple organ failure, and these patients subsequently died.

## Discussion

Penicilliosis is a common but serious opportunistic fungal infection in AIDS patients in Southeast Asia, but is also increasingly observed in HIV-negative individuals. PSM disseminates hematogenously to other locations, with the reticuloendothelial system, skin, and lungs being the most commonly involved sites [6,2]. However, *P. marneffei* infection of the bone leading to osteolytic lesions is extremely rare [3].

In the current retrospective analysis, 100 patients were diagnosed with disseminated PSM, including 65 HIV-infected and 35 HIV-negative patients. All of the 14 patients with osteolytic lesions were HIV-negative, indicating a high incidence rate of PSM with osteolytic lesions of 40% in HIV-negative patients. The presence of *P. marneffei* infection with osteolytic lesions predominantly in HIV-negative patients was consistent with previous reports [7,8]. Thus, despite the importance of osteolytic lesions, they are easily overlooked in HIV-negative patients with *P. marneffei* infection.

Osteolytic lesions primarily occur in HIV-negative patients with *P. marneffei* infection because of their significantly higher white cell population and stronger neutrophil function compared with HIV-positive patients [6,7]. HIV-positive patients also have relatively higher serum antigen titers, while HIV-negative patients have higher serum antibody concentrations, as determined by *P. marneffei*-specific mannoprotein Mp1p ELISA [6]. In HIV-negative patients, when *P. marneffei* disseminates to an osseous site, the significant focal neutrophil aggregation results in localized granulomas and abscesses, leading to significantly increased uptake at osteolytic sites on bone ECT and increased whole-body bone metabolic activity on PET/CT. In contrast, neutrophil function is decreased in immunodeficient HIV-positive patients, and *P. marneffei* infection of osseous sites is therefore rare. There is a prevailing hypothesis that osteolytic lesions typically occur at sites of neutrophil accumulation, where the release of proteolytic enzymes leads to tissue lysis, liquefaction, and necrosis [3].

In our study, PSM with osteolytic lesions could involve any bone, including bones of the trunk, limbs, skull, and tracheal cartilage. The most commonly involved sites were the vertebrae, skull, femur, and ribs, followed by the ilium, clavicle, scapula, humerus, and tibia. The radius, sternum, innominatum, fibula, bones of the hand, humerus, ischium, pubis acetabulum, bones of the foot, sacrum, and cartilage were also involved. Most patients also presented with swelling and tenderness in the soft tissues and joints surrounding the bones. The most striking findings were multiple well-delineated osteolytic lesions, which indicated a predilection for flat bones and long bones, consistent with previous studies [7,8].

The radiographic findings revealed well-circumscribed osteolytic lesions with multiple lucent defects showing a moth-eaten appearance, periosteal proliferation, and fracture. ECT showed significantly increased uptake in many parts of the skeleton, while PET/CT showed increased whole-body bone metabolic activity and diffuse skeletal destruction. ^18^ F-FDG SUV was increased in the entire skeleton, with a mean ^18^ F-FDG SUV of 6.16. Collectively, these findings resemble those of malignancy. Diagnosis is often delayed because penicilliosis marneffei is considered a rare condition in HIV-negative individuals. The radiographic findings usually lag behind the clinical findings by weeks or months, and the variable radiographic presentations and lesion distributions resemble those seen in osteomyelitis caused by tuberculosis, nontuberculous mycobacterial infection, tumors, and other fungal conditions such as African histoplasmosis, blastomycosis, cryptococcosis, and coccidioidomycosis [9-11]. In our study population, osteolytic lesions were a common but easily overlooked finding in HIV-negative individuals with *P. marneffei* infection. *P. marneffei* infection should therefore be considered as one of the common causative organisms in the differential diagnoses of fungal osteomyelitis.

Taking clinical symptoms and signs into consideration, *P. marneffei* osteomyelitis can be diagnosed by its radiological features, typical clinical features, histopathologic examination, and organism culture, specifically from bone, bone marrow, and skin lesions. Bone biopsy with image guidance can increase the positivity rate of fungal cultures.

Penicilliosis is very susceptible to antifungal treatment, and *P. marneffei* is susceptible to itraconazole and amphotericin B *in vitro* [12]. The recommended dosage and duration of treatment among HIV-infected patients are intravenous amphotericin B deoxycholate 0.6–1.0 mg/kg/day for 2 weeks, followed by oral itraconazole 400 mg/day for 8–10 weeks [13]; however, there is currently no recommended treatment for HIV-negative individuals. Six patients died in the current study, representing a high mortality (42.85%), while a further four patients relapsed 3–24 months later despite long-term antifungal treatment, representing a high recurrence rate (28.57%). The patients in this study received antifungal treatment for a longer duration than individuals without osteolysis. There are currently no standard recommendations for the appropriate treatment duration and for prophylaxis of PSM with osteolysis, and treatment depends on the severity of the infection, the response to therapy, and the patient’s immune status [1]. Surgery and external fixation should be performed in patients with persistent or refractory bone disease, such as pathologic fracture and pyogenic osteomyelitis. Our anecdotal experience revealed that relapse was more common in patients who received a shorter duration of treatment. *P. marneffei* involving the bone and leading to osteolysis thus demonstrates the potential of systemic infection to cause severe lesions, and to be associated with poor prognosis and a high recurrence rate, highlighting the need for prolonged antifungal treatment.

## Conclusions

PSM disseminates hematogenously to other locations. However, *P. marneffei* infection of the bone leading to osteolytic lesions is extremely rare. Osteolysis is often overlooked in HIV-negative individuals with disseminated *P. marneffei* infection. However, *P. marneffei* involving the bone and leading to osteolysis may indicate severe systemic disturbance, and is characterized by a poor prognosis, high recurrence rate, and the need for prolonged antifungal treatment.

## Consent

Written informed consent was obtained from all the patients for publication of this report and any accompanying images. A copy of the written consent is available for review by the Editor of this journal.
